# *β*-Sitosterol Alters the Inflammatory Response in CLP Rat Model of Sepsis by Modulation of NF*κ*B Signaling

**DOI:** 10.1155/2021/5535562

**Published:** 2021-04-29

**Authors:** Sara Kasirzadeh, Mohammad Hossein Ghahremani, Neda Setayesh, Fereshteh Jeivad, Amir Shadboorestan, Ali Taheri, Abbas Beh-Pajooh, Armin Azadkhah Shalmani, Alireza Ebadollahi-Natanzi, Alamgir Khan, Samin Sabzevari, Omid Sabzevari

**Affiliations:** ^1^Department of Toxicology and Pharmacology, Faculty of Pharmacy, Tehran University of Medical Sciences, Tehran, Iran; ^2^Toxicology and Poisoning Research Centre, Tehran University of Medical Sciences, Tehran, Iran; ^3^Department of Pharmaceutical Biotechnology, Faculty of Pharmacy and Biotechnology Research Center, Tehran University of Medical Sciences, Tehran, Iran; ^4^Department of Toxicology, Faculty of Medical Sciences, Tarbiat Modares University, Tehran, Iran; ^5^Biopharmaceutics and Pharmacokinetics Division, Department of Pharmaceutics, Faculty of Pharmacy, Tehran University of Medical Sciences, Tehran, Iran; ^6^Medicinal Plants Department, Imam Khomeini Higher Education Center, Agricultural Research, Education and Extension Organization (AREEO), Karaj, Iran; ^7^Australian Proteome Analysis Facility (APAF), Level 4, Building F7B, Research Park Drive, Macquarie University, Sydney, NSW 2109, Australia

## Abstract

**Purpose:**

Sepsis originates from the host inflammatory response, especially to bacterial infections, and is considered one of the main causes of death in intensive care units. Various agents have been developed to inhibit mediators of the inflammatory response; one prospective agent is *β*-sitosterol (*β*S), a phytosterol with a structure similar to cholesterol. This study is aimed at evaluating the effects of *β*S on the biomarkers of inflammation and liver function in cecal ligation and puncture- (CLP-) induced septic rats.

**Methods:**

Thirty male Wistar rats were divided equally into six groups as follows: sham, CLP, CLP+dexamethasone (DX, 0.2 mg/kg), CLP+*β*S (1 mg/kg), CLP+imipenem (IMI, 20 mg/kg), and CLP+IMI (20 mg/kg)+*β*S (1 mg/kg). Serum levels of IL-1*β*, IL-6, IL-10, AST, ALT, and liver glutathione (GSH) were assessed by ELISA. Liver expression levels of TNF-*α* and NF-*κ*Bi mRNAs were evaluated by RT-qPCR.

**Results:**

Serum concentrations of IL-1*β*, IL-6, IL-10, ALT, and AST and mRNA levels of TNF-*α* and NF-*κ*Bi were all significantly higher in septic rats than in normal rats (*p* < 0.05). Liver GSH content was markedly lower in the CLP group than that in the sham group. *β*S-treated rats had remarkably lower levels of IL-1*β*, IL-6, IL-10, TNF-*α*, NF-*κ*Bi, AST, and ALT (51.79%, 62.63%, 41.46%, 54.35%, 94.37%, 95.30%, 34.87%, and 46.53% lower, respectively) and greater liver GSH content (35.71% greater) compared to the CLP group (*p* < 0.05).

**Conclusion:**

*β*S may play a protective role in the septic process by mitigating inflammation. This effect is at least partly mediated by inhibition of the NF-*κ*B signaling pathway. Thus, *β*S can be considered as a supplementary treatment in septic patients.

## 1. Introduction

Sepsis is a life-threatening critical illness caused by the invasion of microbial pathogens into the bloodstream. It remains one of the major causes of death specifically in intensive care units. According to the World Health Organization (WHO), sepsis affects more than 30 million people each year, causing approximately six million deaths worldwide [1]. Excessive and uncontrolled response to infection, septic shock, and acute failure of one or multiple organs such as the liver, kidney, and lungs can occur and may result in death in sepsis patients [2, 3]. It has been reported that liver dysfunction is a subsequent result of sepsis, confirmed by higher serum levels of aspartate aminotransferase (AST) and alanine aminotransferase (ALT), and lower glutathione (GSH) content [3].

**Figure 1:**
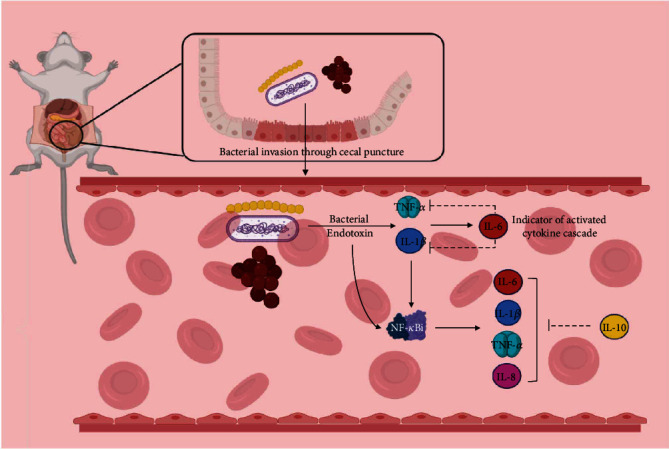
Cytokine cascade after CLP induction. Invasion of stimuli (bacterial endotoxin) to the blood is the trigger of cytokine cascade. An increase in the early proinflammatory elements (such as IL-1*β* and TNF-*α*) results in the production of other inflammatory factors such as NF-*κ*B, IL-6, and anti-inflammatory cytokines such as IL-10. An uncontrolled cytokine cascade will lead to sepsis and, in severe cases, to septic shock. Tissue/organ dysfunction or end-organ damage might occur following progressive cytokine imbalance. CLP: cecal ligation and puncture; IL: interleukin; TNF: tumor necrosis factor.

Cytokines, defined as a group of endogenous inflammatory and immune-modulating proteins, play a dominant role in sepsis syndrome. Exacerbated production of both pro- and anti-inflammatory cytokines results in a situation called “cytokine cascade.” A cytokine cascade is initiated when a host is targeted by stimuli such as bacterial endotoxins, which induce the production of early cytokines including tumor necrosis factor-alpha (TNF-*α*) and interleukin 1-beta (IL-1*β*). Overproduction of early cytokines has been associated with multiple end-organ dysfunction and mortality [4]. Along with endotoxins, these cytokines are able to stimulate the production of later cytokines, such as interleukin 6 (IL-6). Interestingly, IL-6 is responsible for the downregulation of TNF-*α* and IL-1*β*, which might be vital in limiting the cytokine cascade [2]. Nuclear factor-kappa B (NF-*κ*B) also plays a regulatory role in the cytokine cascade and is activated in numerous cell types by endotoxins, TNF-*α*, and IL-1*β*. Activation of NF-*κ*B is a pivotal step for the transcription of many cytokines including TNF-*α*, IL-1*β*, IL-6, and IL-8. However, as activation of NF-*κ*B increases, the transcription of NF-*κ*B inhibitor (NF-*κ*Bi) also rises. The protein product of NF-*κ*Bi, I*κ*B*α*, is an indicator of NF-*κ*B activation. Newly synthesized I*κ*B*α* translocates into the nucleus and terminates NF-*κ*B activation by binding to the activated dimer. NF-*κ*Bi level increases in response to inflammation and decreases when appropriate treatment is given [5]. Furthermore, the NF-*κ*B signaling pathway has been reported to play a role in hepatic injury [6]. In addition to the cytokine cascade, several studies have demonstrated that the balance between pro- and anti-inflammatory cytokines is a major factor determining the severity of sepsis [7, 8]. *In vitro* studies have shown that the anti-inflammatory cytokine interleukin 10 (IL-10) is a regulatory factor that blocks TNF-*α*, IL-1*β*, and IL-8 [4] and many studies have found higher concentrations of IL-10 in patients with sepsis [9, 10] (Figure 1).

Many natural plants contain pharmacologically active compounds that possess immunomodulating and anti-inflammatory properties and can potentially inhibit the cytokine cascade [11]. *β*-Sitosterol (*β*S) is a phytosterol found in various plants and has a chemical structure similar to that of cholesterol [12]. Several *in vitro* studies have revealed *β*S to possess anti-inflammatory properties. *β*S has been shown to have antiatherogenic potential through its anti-inflammatory action on human aortic endothelial cells [13]. Similarly, it has been reported that *β*S causes a dose-dependent inhibition of IL-6 and TNF-*α* in endotoxin-activated human monocytes [13, 14]. It has been found that *β*S has beneficial effects on the immune system by increasing the number of viable peripheral blood mononuclear cells (PBMCs) and activating the swine dendritic cells (DCs) [15]. Moreover, *β*S has been evaluated as an anti-inflammatory agent in a number of animal models in *in vivo* experiments. Anti-inflammatory and immunomodulatory properties of *β*S have been demonstrated in several animal models, including fattener pigs after receiving a modified live porcine reproductive and respiratory syndrome virus (PRRSV) vaccine [15], rats with oxazolone-induced contact-delayed type hypersensitivity [16], and mice with ovalbumin-induced lung inflammation [17]. Clinical studies represented the potential immunomodulatory effects of *β*S in patients with pulmonary tuberculosis, human immunodeficiency virus (HIV), human papillomavirus (HPV), stress-induced immune suppression, rheumatoid arthritis, and allergic rhinitis and sinusitis [14].

Our research is aimed at evaluating the effects of *β*S administration on the biomarkers of inflammation and liver function in cecal ligation and puncture (CLP) rats as a gold standard animal model of sepsis.

## 2. Methods

### 2.1. Animals

Thirty male Wistar rats, weighing 210-250 g, were acquired from the Animal House, Faculty of Pharmacy, Tehran University of Medical Sciences, Tehran, Iran. The animals were housed in a temperature-controlled environment (25 ± 3°C) on a 12 h light/dark cycle (lights on at 08:00 AM) and were provided with water and standard diet ad libitum. The rats did not fast before CLP surgery (Section 2.3.). All animals were acclimated to the laboratory environment for seven days before surgery. Experimental procedures were conducted according to the Institutional Animal Care and Use Committee (IACUC) guidelines and were approved by the Animal Ethics Committee of Tehran University of Medical Sciences, Tehran, Iran (Code: IR.TUMS.REC.1394.1837).

### 2.2. Study Design

Animals were randomly divided into six groups of five rats each. The first group was the sham group and received 5 mL normal saline (NS) as fluid resuscitation at 6 h and 24 h after sham surgery. CLP surgery was performed in all other groups. The second group (CLP group) received NS at 6 h and 24 h after surgery. The third group (DX group) received NS and dexamethasone (DX, 0.2 mg/kg) at 6 h and 24 h after surgery. The fourth group (*β*S group) received NS and *β*S (1 mg/kg) at 6 h and 24 h after surgery. The fifth group (IMI group) received NS and imipenem reconstituted in NS (IMI, 20 mg/kg) at 6 h, 24 h, 36 h, and 48 h after surgery. The sixth group (IMI+*β*S group) received NS, IMI (20 mg/kg), and *β*S (1 mg/kg) at 6 h, 24 h, 36 h, and 48 h after surgery. DX and IMI were obtained from 13Aban Pharmacy (Tehran, Iran), and *β*S was purchased from Zardband Company (Tehran, Iran), with 95.41% purity as assessed by high-performance liquid chromatography at 208 nm by the manufacturer (Zardband Company, Tehran, Iran). All animals in the sham, CLP, DX, and *β*S groups were euthanized 48 h after surgery, and those in the IMI and IMI+*β*S groups were euthanized 72 h after surgery by ketamine (160 mg/kg) and xylazine (20 mg/kg). Liver tissue was removed and washed thoroughly with cold-sterile phosphate-buffered saline (PBS) to remove blood residues. It was then snap-frozen in liquid nitrogen for real-time quantitative polymerase chain reaction (RT-qPCR).

### 2.3. CLP

The animals were anesthetized under aseptic conditions by intraperitoneal (i.p.) administration of ketamine (80 mg/kg) and xylazine (10 mg/kg). A 2 cm midline laparotomy was performed longitudinally and medially on the anterior abdomen. The distal 20% was ligated by 3.0 silk sutures below the ileocecal valve to make a closed-loop and was perforated two times with a sterile 21-gauge needle. The loop (cecum) was manually and gently squeezed to extrude fecal material from the punctures, and then placed back into the abdominal cavity. The peritoneum and skin were sutured in two layers. After surgery, all groups were resuscitated with 5 mL NS by subcutaneous injection. A thermoregulated heating pad and overhead heating lamp were used to maintain core body temperature (37°C). The sham group underwent surgery identical to CLP, with the exception that ligation and puncture procedures were not performed.

### 2.4. Bicinchoninic Acid Assay (BCA)

Total amounts of serum and liver tissue protein were assayed using a BCA protein quantification kit (ParsTous, Tehran, Iran) according to the manufacturer's instructions. Briefly, one part of copper reagent was added to 50 parts of BCA reagent to prepare the working solution. The serial dilutions of bovine serum albumin (BSA) standard were used to prepare the standard curve. Standards and sample solutions were pipetted into wells. The working solution was added into all the wells. The wells were incubated for 60 minutes at 60°C. Optical density (OD) was measured at 562 nm using an enzyme-linked immunosorbent assay (ELISA) plate reader (BioTek, Winooski, VT, USA). The blank OD was subtracted from all standard and sample OD values. Each sample protein concentration was determined using the standard curve.

### 2.5. Assessment of Liver Damage

Serum levels of AST and ALT were determined using commercially available ELISA kits (Pars Azmoon Inc., Tehran, Iran) and according to the manufacturer's instructions. The serum ALT levels were measured based on two parallel reactions. Firstly, ALT transfers the amino group of alanine to *α*-ketoglutaric acid. The products of this reaction are pyruvate and glutamate. Then, lactate dehydrogenase converts pyruvate to lactate while oxidizing NADH to NAD^+^. This oxidation results in a decrease in absorbance at 340 nm, and this reduction is proportional to the ALT level in the sample [18]. Relatively similar reactions were used to measure the serum AST levels. AST transfers the amino group of aspartate to *α*-ketoglutaric acid to form oxaloacetate and glutamate. Then, malate dehydrogenase changes oxaloacetate to malate while oxidizing NADH to NAD^+^ [19]. The absorbance was measured using an ELISA plate reader (BioTek, Winooski, VT, USA).

### 2.6. GSH Content

GSH levels were determined using Ellman's reagent (4 mg DTNB [5,5′-dithiobis-(2-nitrobenzoic acid)] in 10 mL sodium citrate 10%) [20]. In brief, equal volumes of 10% trichloroacetic acid were added to liver homogenates. The mixtures were then vortexed and centrifuged at 15,000 *g* for 10 min at 4°C. Following centrifugation, 100 *μ*L of the resulting supernatants was added to a 96-well microplate. Subsequently, 200 *μ*L of Ellman's reagent was added to each well. The absorbance was measured at 412 nm using an ELISA plate reader (BioTek, Winooski, VT, USA).

### 2.7. Detection and Quantitation of Cytokines

Following euthanasia, blood samples were collected in sterile blood-collecting tubes by cardiac puncture and allowed to clot for 2 h at room temperature. Following the protocol, serum was removed and stored at ≤-20°C. All samples were centrifuged for 20 minutes at 1000 *g*. IL-1*β*, IL-6, and IL-10 concentrations were then measured using ELISA kits specific for rats (R&D Systems, Minneapolis, MN, USA). All reagents, standard dilutions, controls, and samples were prepared according to the manufacturer's instructions. 50 *μ*L of assay diluent (specific for each cytokine) was added to each well. Then, 50 *μ*L of standards, controls, or samples was added to each well. The plates were covered with an adhesive strip and incubated for 2 h at room temperature. The standards, controls, and samples were recorded on a plate layout. Each well was aspirated and washed five times by filling with wash buffer (400 *μ*L). After removing any remaining wash buffer, the plates were inverted and blotted against clean paper towels. 100 *μ*L of rat IL-1*β*, IL-6, or IL-10 was added to each well. The plates were covered with an adhesive strip and incubated for another 2 h at room temperature. Each well was aspirated and washed five times as previously described. 100 *μ*L of substrate solution was added to each well and incubated at room temperature for 30 minutes away from light. For the last step, 100 *μ*L of stop solution was added to each well, and after 30 minutes, the ODs were determined at 450 nm and 540 nm. The readings at 540 nm were subtracted from 450 nm to correct optical imperfections in the plates. Spectrophotometric readings were taken using the BioTek Synergy 4 microplate reader (BioTek, Winooski, VT, USA). Data obtained from ELISA were then normalized to the total protein in each serum sample, which was measured by the BCA method (Section 2.4).

### 2.8. Selection of Reference and Target Genes and Design

GAPDH has been shown to be a suitable reference gene for use in normalizing liver tissue gene expression values obtained through RT-qPCR [21]. DNA sequences of GAPDH and target genes were obtained from the GenBank database, and corresponding primer sequences (Table 1) were designed using Primer-BLAST from NCBI. The primers were designed in exon-exon junctions to minimize genomic DNA contamination.

Primers were synthesized at the highest quality (Sinaclon, Tehran, Iran), and primer specificity was confirmed by melt curve analysis. RT-qPCR products were evaluated by electrophoresis in a 2% agarose gel to confirm the quality of products.

### 2.9. RT-qPCR

The expression of TNF-*α* and NF-*κ*Bi mRNA was determined by RT-qPCR. RNA samples were extracted from liver tissue following the TriPure isolation reagent protocol (Roche, Basel, Switzerland). RNA OD was measured using Eppendorf BioPhotometer (Eppendorf, Hamburg, Germany). RNA extracts were then used as templates for cDNA synthesis. Considering obtained RNA OD values, cDNA synthesis was performed with the PrimeScript™ RT reagent kit (Takara Biotechnology, Shiga, Japan) according to the manufacturer's instructions, using 5 *μ*L of RNA (corresponding to 1 *μ*g), 10 *μ*L of RT-premix, 1 *μ*L of random hexamers, and 4 *μ*L of DEPC-RNase/DNase-free water. The primer sequences used in this study are listed in Table 1. RT-qPCR was conducted using Applied Biosystems StepOnePlus (Thermo Fisher, Waltham, MA, USA) and the SYBR Premix Ex Taq™ II kit (Takara Biotechnology, Shiga, Japan). Reactions were prepared in a total volume of 10 *μ*L, each containing 3 *μ*L of template cDNA (corresponding to 1 *μ*g RNA), 0.5 *μ*L of each primer (forward and reverse with concentrations equal to 5 pmol/*μ*L), 5 *μ*L of SYBR Green master mix, 0.2 *μ*L of ROX reference dye, and 0.8 *μ*L of RNase/DNase-free sterile water. After 5 min of room temperature incubation, the cDNA synthesis cycle (42°C for 90 min, followed by 95°C for 10 min) was performed on an Applied BioSystems 96-well thermal cycler. PCR was conducted with an initial single heating cycle (95°C for 10 min) followed by 45 amplification cycles (95°C for 5 s, 60°C for 40 s). All samples were analyzed in a single analytical run (RT-qPCR reaction) to exclude any potential variation. Finally, for each primer pair, product specificity and the absence of primer dimer formation were confirmed using a dissociation protocol with a heat gradient ranging from 60°C to 95°C. In order to control DNA contamination, no-template control (NTC) reactions were included in each experiment. Reactions were performed three times for each gene, and the mean value was used for statistical analyses using Applied Biosystems StepOnePlus (Thermo Fisher, Waltham, MA, USA). GAPDH was used as the housekeeping gene. The expression levels of TNF-*α* and NF-*κ*Bi mRNA were normalized to that of GAPDH as an internal control. All data were then normalized to the sham group [22, 23].

### 2.10. Statistical Analysis

GraphPad Prism version 8 for Windows was used for statistical analysis. Data are presented as the mean ± standard deviation (SD). One-way analysis of variance (ANOVA) was used to compare the sham, CLP, DX, and *β*S groups 48 h postsurgery. Post hoc comparisons were made with Tukey's post hoc test. Unpaired *t*-test was used to compare the IMI and IMI+*β*S groups 72 h postsurgery. The Kaplan-Meier method was used to estimate survival rates at 48 h postsurgery, and the log-rank test was used to compare the study groups. Significance was defined as *p* < 0.05.

## 3. Results

### 3.1. Serum Levels of AST and ALT

**Figure 2:**
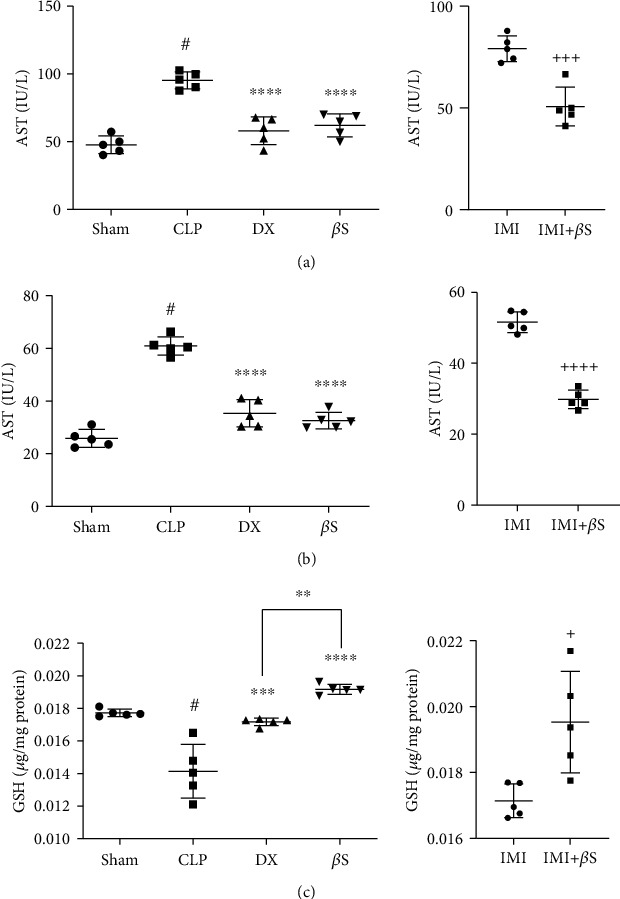
Measurement of AST (a) and ALT (b) serum levels and GSH content of liver tissue (c) using ELISA (*n* = 5/group). Measurements were performed 48 h after surgery in the sham, CLP, DX, and *β*S groups, and 72 h after surgery in the IMI and IMI+*β*S groups. All data are expressed as the mean ± SD. Data were analyzed by ANOVA, followed by Tukey's post hoc test for sham, CLP, DX, and *β*S comparison. The IMI and IMI+*β*S groups were analyzed separately using unpaired *t*-test. ^#^Significant difference compared with the sham group (*p* < 0.0001). ^∗∗^Significant difference compared with the DX group (*p* < 0.01). ^∗∗∗^Significant difference compared with the CLP group (*p* < 0.001). ^∗∗∗∗^Significant difference compared with the CLP group (*p* < 0.0001). ^+^Significant difference compared with the IMI group (*p* < 0.05). ^+++^Significant difference compared with the IMI group (*p* < 0.001). ^++++^Significant difference compared with the IMI group (*p* < 0.0001). *β*S: *β*-sitosterol; CLP: cecal ligation and puncture; DX: dexamethasone; IMI: imipenem.

AST and ALT are effective modalities and biomarkers of liver function. The CLP group demonstrated significantly higher levels of both AST and ALT (*p* < 0.0001) in comparison to the sham group (Figures 2(a) and 2(b)). Both AST and ALT levels were markedly lower in groups that were treated with either DX or *β*S (*p* < 0.0001). ALT level was lower in the *β*S group than in the DX group; however, the difference was not statistically significant. The addition of *β*S to IMI (IMI+*β*S) resulted in markedly lower levels of AST and ALT compared with IMI-only treatment (*p* < 0.001 and *p* < 0.0001, respectively).

### 3.2. GSH Content of Liver Tissue

GSH is a pivotal antioxidant that acts as a scavenger of reactive oxygen species (ROS). GSH content was assessed as an indicator of oxidative stress. Significant depletion of GSH was observed in the CLP group compared to the sham group (*p* < 0.0001, Figure 2(c)). GSH content was higher in the DX- and *β*S-treated groups compared to the CLP group (*p* < 0.001 and *p* < 0.0001, respectively). GSH level was higher in *β*S-treated rats compared with the DX group (*p* < 0.01). Moreover, the addition of *β*S to IMI (IMI+*β*S group) resulted in significantly higher levels of GSH (*p* < 0.05).

### 3.3. Serum Concentrations of IL-1*β*, IL-6, and IL-10

**Figure 3:**
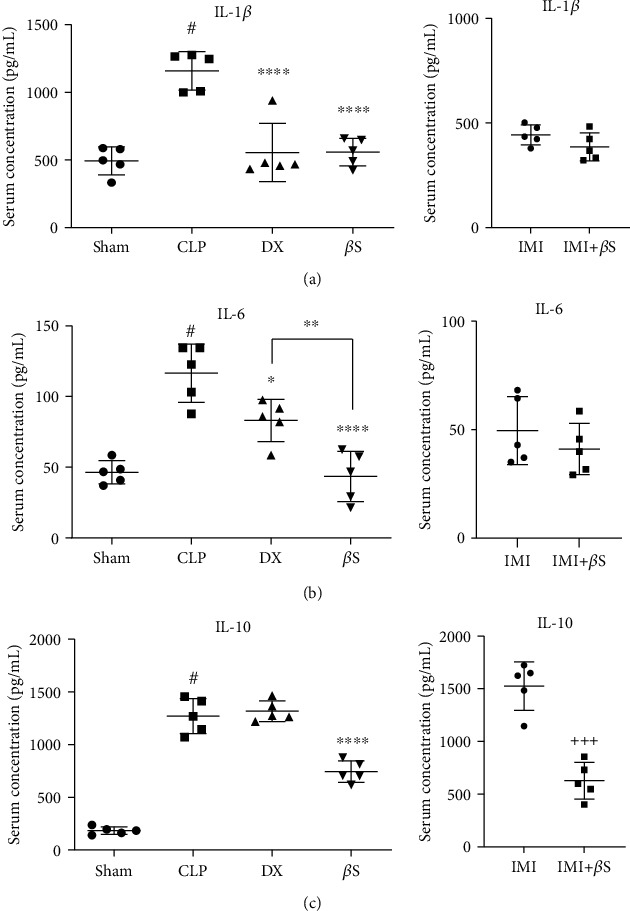
Measurement of IL-1*β* (a), IL-6 (b), and IL-10 (c) serum levels using ELISA (*n* = 5/group). Measurements were performed 48 h after surgery in the sham, CLP, DX, and *β*S groups, and 72 h after surgery in the IMI and IMI+*β*S groups. All data are normalized to the total protein levels measured by BCA assay (mean ± SD of 0.205 ± 0.0001, 0.205 ± 0.0001, 0.204 ± 0.0001, 0.204 ± 0.0001, 0.204 ± 0.0001, and 0.205 ± 0.0003 pg/mL in the sham, CLP, DX, *β*S, IMI, and IMI+*β*S groups, respectively), and expressed as the mean ± SD. Data were analyzed by ANOVA, followed by Tukey's post hoc test for sham, CLP, DX, and *β*S comparison. The IMI and IMI+*β*S groups were analyzed separately using unpaired *t*-test. ^#^Significant difference compared with the sham group (*p* < 0.0001). ^∗^Significant difference compared with the CLP group (*p* < 0.05). ^∗∗^Significant difference compared with the DX group (*p* < 0.01). ^∗∗∗∗^Significant difference compared with the CLP group (*p* < 0.0001). ^+++^Significant difference compared with the IMI group (*p* < 0.001). *β*S: *β*-sitosterol; CLP: cecal ligation and puncture; DX: dexamethasone; IMI: imipenem.

Rats in the CLP group had remarkably higher serum levels of IL-1*β*, IL-6, and IL-10 compared with the sham group (*p* < 0.0001). Treatment with *β*S resulted in considerably lower levels of these cytokines compared with the CLP group (IL-1*β*, *p* < 0.0001; IL-6, *p* < 0.0001; and IL-10, *p* < 0.0001) (Figure 3). DX-treated rats showed lower levels of IL-1*β* and IL-6 compared with the CLP group (*p* < 0.0001 and *p* < 0.05, respectively). *β*S treatment resulted in considerably lower serum IL-6 levels as compared with DX treatment (*p* < 0.01). Although the differences in the levels of IL-1*β* and IL-6 between the IMI and IMI+*β*S groups were not statistically significant (*p* > 0.05), IL-10 levels were significantly lower in IMI+*β*S compared with the IMI group (*p* < 0.001).

### 3.4. Liver Expression of TNF-*α* and NF-*κ*Bi

**Figure 4:**
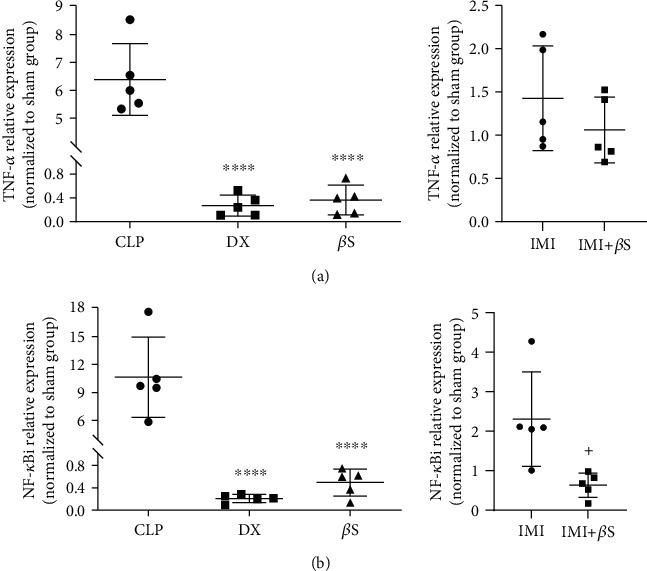
mRNA expression of TNF-*α* and NF-*κ*Bi in liver tissue. TNF-*α* and NF-*κ*Bi mRNA expression was assessed using predeveloped assays for RT-qPCR. Values were calculated using a comparative CT method (2^−ΔΔCT^) according to the manufacturer's instructions. GAPDH was used as an internal control gene. Data are presented as the mean ± SD normalized to the sham group. Statistical analysis was performed using one-way ANOVA and Tukey's multiple comparisons test for sham, CLP, DX, and *β*S comparison. The IMI and IMI+*β*S groups were analyzed separately using unpaired *t*-test. ^∗∗∗∗^Significant difference compared with the CLP group (*p* < 0.0001). ^+^Significant difference compared with the IMI group (*p* < 0.05). *β*S: *β*-sitosterol; CLP: cecal ligation and puncture; DX: dexamethasone; IMI: imipenem.

Liver levels of TNF-*α* and NF-*κ*Bi mRNAs were determined by RT-qPCR. Higher concentrations of TNF-*α* and NF-*κ*Bi mRNAs were observed in the CLP group (Figure 4; data are presented relative to the sham group). Treatment with DX (*p* < 0.0001) and *β*S (*p* < 0.0001) resulted in remarkably lower TNF-*α* expression levels compared with the CLP group (Figure 4(a)). IMI+*β*S treatment led to lower levels of TNF-*α* mRNA compared with the IMI group, but the difference was not statistically significant. DX (*p* < 0.0001) and *β*S (*p* < 0.0001) treatments resulted in significantly lower levels of NF-*κ*Bi mRNA compared with the CLP group, and interestingly, the addition of *β*S to IMI led to significantly lower levels of NF-*κ*Bi mRNA (*p* < 0.05) compared with IMI-only treatment (Figure 4(b)).

### 3.5. Survival Rates

**Figure 5:**
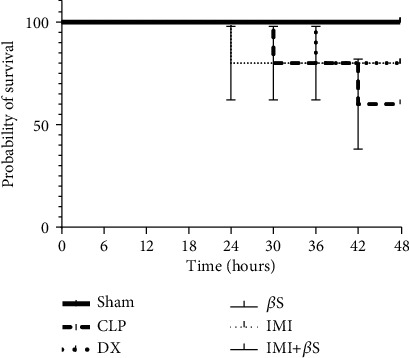
Survival rates over the 48 h period after surgery (observations every 6 h). There was no statistically significant difference in survival rates between the study groups (*p* > 0.05). *β*S: *β*-sitosterol; CLP: cecal ligation and puncture; DX: dexamethasone; IMI: imipenem.

Survival rates were compared using the Kaplan-Meier method (Figure 5). The survival rates were 60% (3/5 rats) in the CLP group and 80% (4/5 rats) in the DX and IMI groups by day two. Survival rates in the sham, *β*S, and IMI+*β*S groups were 100% 48 h after surgery. According to the log-rank (Mantel-Cox) test, the survival curves were not significantly different between groups (*p* > 0.05).

## 4. Discussion

Inflammatory responses are believed to play a critical role in the underlying mechanisms of inflammatory diseases like sepsis. Hence, a combination of anti-inflammatory drugs and antibiotics is recommended to improve sepsis severity and prognosis [24]. Here, IMI was used as the antibiotic agent, as it has a broad-spectrum antibacterial activity [25, 26]. DX, a glucocorticoid drug that is widely used for sepsis patients, was also considered [24]. The physiological roles of phytosterols in the inflammatory processes have yet to be elucidated. Therefore, in the present study, we aimed to evaluate the association between *β*S administration and inflammatory cytokines by conducting an in vivo research using the gold standard method of sepsis induction, CLP.

Our study provides considerable insight into the anti-inflammatory properties of *β*S. We observed lower levels of IL-1*β*, IL-6, and IL-10 serum levels in the *β*S-treated group compared with the CLP group. Although adding *β*S to IMI treatment did not significantly alter IL-1*β* and IL-6 levels, it resulted in a remarkable decrease in IL-10 serum level compared with IMI alone, suggesting that *β*S might help to control the inflammation caused by cytokine cascade via modulation of anti-inflammatory cytokines. Furthermore, *β*S treatment caused a significantly greater reduction in IL-6 level compared with DX. Glucocorticoids such as DX are proven anti-inflammatory agents in sepsis management. Glucocorticoid therapy interferes with the production of various proinflammatory cytokines such as IL-6. Schmidt et al. [27] reported that DX administration decreased IL-6 production in a CLP-induced sepsis study. Given this context, the statistically distinct reduction of IL-6 by *β*S supports the potential usefulness of *β*S in sepsis-induced inflammation. Our observations on the effects of *β*S on inflammatory cytokines are in agreement with several other studies. One recent study suggested a negative correlation between *β*S and IL-6 levels in an animal model of obesity-related chronic inflammation [28]. Another study revealed a decrease in IL-1*β* levels in murine-activated neutrophils following *β*S therapy [29]. However, our data on the effects of *β*S on serum levels of IL-6 and IL-10 is not in agreement with a study conducted by Alappat et al. [30] on the immune function of macrophages. Their findings demonstrated higher serum concentrations of IL-6 and IL-10 in the *β*S group compared with the sham group. These contradictory results might be due to different dosages, times of sampling, and the type of experimental study (i.e., *in vitro* vs. *in vivo*). Although IL-10 is an anti-inflammatory mediator and could potentially suppress the cytokine cascade, the higher levels may lead to organ dysfunction, as previously confirmed by other studies [31, 32].

Rats treated with *β*S showed lower expression levels of TNF-*α* mRNA and NF-*κ*Bi mRNA in the liver compared with the CLP group, which is in line with the IL-1*β* and IL-6 lower levels in serum samples. *β*S also exerts beneficial effects on the cytokine cascade by targeting NF-*κ*B. The inhibition of NF-*κ*B by *β*S has been extensively investigated, with published findings that are in line with our data [13, 33, 34]. The immunoblot and confocal analyses of lipopolysaccharide- (LPS-) stimulated intestinal and peritoneal mouse macrophages by Kim et al. [33] showed that *β*S inhibits the phosphorylation and nuclear translocation of the p65 subunit of NF-*κ*B. Similarly, ImageStream cytometry analysis by Valerio and Awad [34] showed that 24 h treatment of mouse macrophages with 4 *μ*M *β*S and stimulation with LPS for the last 6 h resulted in 45% lower translocation of NF-*κ*B to the nucleus compared to the LPS alone. The stabilization of NF-*κ*Bi mRNA by *β*S is believed to be the mechanism underlying TNF-*α* downregulation, reducing its phosphorylation, and consecutively depleting the nuclear NF-*κ*B complex [35]. The most striking result that emerges from our data is that the administration of *β*S and DX both resulted in significantly lower levels of TNF-*α* and NF-*κ*Bi mRNA expression compared to the CLP group (*p* < 0.0001), and the level of expression was the same between the *β*S and DX groups as confirmed by Tukey's multiple comparisons test (*p* > 0.05). This further supports that *β*S could be considered as an effective intervention for controlling sepsis-induced inflammation.

Liver injury is common among sepsis patients. The incidence of liver dysfunction varies in different studies from 34% to 46% [36]. Along with the sepsis-associated liver injury, other liver injuries such as diseases caused by hepatitis B and C viruses, alcoholic liver disease, nonalcoholic fatty liver disease, and drug-induced injuries are also common among sepsis patients [37]. Elevation of serum AST and ALT and reduction of liver GSH are signs of liver malfunction [3]. In our experiment, compared with the sham group, CLP rats had significantly higher levels of AST and ALT. These levels were markedly lower in the *β*S-treated rats. Similarly, compared with the sham group, liver GSH content was significantly lower in the CLP group, and *β*S treatment restored the GSH content. Furthermore, the level of GSH in the *β*S group was significantly greater than that in the DX group. The addition of *β*S to IMI (IMI+*β*S group) also resulted in significantly lower levels of ALT and significantly higher levels of liver GSH content compared to the IMI-only group. These results suggest that *β*S could provide advantageous effects on liver function and also demonstrate its possible antioxidant activity. Our data confirm the results provided by Wong et al. [38], which demonstrated the favorable effects of *β*S against carbon tetrachloride-induced hepatotoxicity with significant improvements in AST, ALT, and GSH levels. Liver dysfunction during sepsis is associated with an increased risk of multiple organ failure and death. Mortality occurs in up to 68% of sepsis patients with liver dysfunction [36]. Data from our study, showing reduced serum levels of AST and ALT, suggest that *β*S may improve liver function in sepsis. Redox imbalance and severe oxidative stress occur in critically ill sepsis patients and can cause multiple organ failure [39, 40]. Glutathione is the primary antioxidant in cells, meaning that the lower the glutathione content, the greater the risk of organ failure becomes [41]. Thus, increased liver GSH content with *β*S treatment indicates that it may improve prognosis and limit liver dysfunction in sepsis.

We additionally investigated the survival rates over the 48 h period after treatment (observed every 6 h). CLP-induced rats exhibited 60% survival. All animals in the sham, *β*S, and IMI+*β*S groups survived up to 48 h, while the IMI and DX groups had a lower survival rate of 80%. Based on the Kaplan-Meier curves, these differences in survival rates were not statistically significant. Our data do not allow drawing any clear conclusions about the impact of *β*S on survival rate.

Doğan et al. [42] evaluated the antibacterial effects of different steroid substances. While they did not find any significant antibacterial activity for progesterone, estrone, and stigmasterol, they reported that *β*S inhibits the growth of *Staphylococcus aureus* with a minimum inhibitory concentration (MIC) value of 32 *μ*g/mL. In another study by Sen et al. [43], the antibacterial activity of *β*S was examined using the agar disk diffusion method. *β*S was found to be effective against *Escherichia coli*, *Pseudomonas aeruginosa*, *Staphylococcus aureus*, and *Klebsiella pneumoniae*. They reported that the antibacterial activity of *β*S (20 *μ*g/mL) was almost equivalent to that of gentamicin (20 *μ*g/mL). Similarly, Burčová et al. [44] demonstrated the bacteriostatic effects of *β*S on *Escherichia coli*, *Bacillus cereus*, *Pseudomonas aeruginosa*, *Bacillus subtilis*, and *Listeria monocytogenes*. A wide range of bacteria were affected by *β*S in these studies, and this can potentially provide additional benefits for sepsis patients. More pharmacokinetic and pharmacodynamic studies are required to fully evaluate the antibacterial properties of *β*S in sepsis.

Here, we demonstrated the beneficial impacts of *β*S administration on inflammatory responses and liver function tests in septic rats. By dampening the hyperinflammatory damage, *β*S may provide some benefits in diseases with molecular patterns similar to sepsis, such as the coronavirus disease 2019 (COVID-19). It has been demonstrated that the extensive release of inflammatory mediators such as TNF-*α*, IL-6, and IL-10 is associated with increased mortality risk in COVID-19 patients [45]. Moreover, this cytokine storm may lead to liver dysfunction in these patients as elevated AST/ALT levels are widely reported in the literature [46]. The potential benefits of *β*S supplementation in COVID-19 could be investigated in future research. The disturbance of immune cells, including a drastic depletion of CD4 and CD8 lymphocytes, occurs in sepsis patients [47]. The evaluation of the effects of *β*S on the status of immune cells is warranted in future studies.

## 5. Conclusions

Overproduction of proinflammatory cytokines contributes to high mortality rates in sepsis patients. In our study, we observed that treatment with *β*S at a dose of 1 mg/kg for two days results in a significant reduction of proinflammatory cytokines in the sera of CLP-induced septic rats. Liver expression of TNF-*α* and NF-*κ*Bi was also reduced in rats treated with *β*S. Similarly, the addition of *β*S to IMI led to an improvement in the serum and liver inflammatory markers compared to IMI alone at three days after sepsis induction. We concluded that *β*S has noticeable potential for consideration as an anti-inflammatory supplement in hyperinflammatory conditions such as sepsis. Our findings would seem to suggest that *β*S targets NF-*κ*B as a major regulator of cytokine cascade and inhibits inflammatory response to a great extent. Liver dysfunction is common among sepsis patients and is associated with a poor prognosis. Here, we found that *β*S treatment effectively lowered the levels of serum biomarkers of liver dysfunction (AST and ALT) and increased the liver GSH content in septic rats. Thus, *β*S could alleviate liver oxidative stress and could inhibit possible organ dysfunction. The results so far are encouraging and promising, and this could eventually lead to a better understanding of the beneficial effects of *β*S against hyperinflammation. Future research should provide further insight into the potential use of *β*S in sepsis and similar clinical conditions with systemic inflammation.

## Figures and Tables

**Table 1:** Reference and target genes and the corresponding primers used in RT-qPCR.

Symbol	Gene name	Mechanism	GenBank ID	Primer sequences	Amplicon length (bp)
GAPDH	Glyceraldehyde-3-phosphate dehydrogenase	Glycolytic enzyme	NM 017008	FP: 5′-AGTGCCAGCCTCGTCTCATA-3′ RP: 5′-GGTAACCAGGCGTCCGATA-3′	77
TNF-*α*	Tumor necrosis factor-alpha	Proinflammatory cytokine	NM 012675	FP: 5′-TGGGCTCCCTCTCATCAGTT-3′ RP: 5′-CTTGGTGGTTTGCTACGACG-3′	104
NF-*κ*Bi	Nuclear factor kappa B inhibitor-alpha	Inhibitory enzyme	NM001105720	FP: 5′-GTGACTTTGGGTGCTGATGT-3′ RP: 5′-ACACTTCAACAGGAGCGAGA-3′	111

bp: base pair; FP: forward primer; RP: reverse primer.

## Data Availability

The data supporting the conclusions of this study are available from the corresponding author upon request.
